# Transverse incisions improve scar outcomes in anterolateral supine approach total hip arthroplasty: a patient observer scar assessment scale-based study

**DOI:** 10.1186/s42836-025-00354-7

**Published:** 2026-01-05

**Authors:** Yujiro Kuramitsu, Junya Itou, Yutaro Munakata, Ken Okazaki

**Affiliations:** Department of Orthopaedic Surgery, Tokyo Women’s Medical University, 8-1 Kawada-Cho, Shinjuku, Tokyo 162-8666 Japan

**Keywords:** Total hip arthroplasty, Patient observer scar assessment scale, Transverse incision, Longitudinal incision, Anterolateral supine approach

## Abstract

**Background:**

This study compared transverse and longitudinal skin incisions in anterolateral supine (ALS) total hip arthroplasty (THA), focusing on cosmetic and sensory outcomes using the Patient Observer Scar Assessment Scale (POSAS).

**Methods:**

A retrospective analysis was conducted on 132 hips that underwent primary ALS THA performed by a single surgeon between 2019 and 2024. Longitudinal incisions were used until December 2022, and transverse incisions aligned with relaxed skin tension lines were used thereafter. POSAS 3.0 was used to evaluate scar quality across satisfaction, appearance, and sensory domains.

**Results:**

Baseline characteristics were similar between groups, except for follow-up duration and incision length. No significant differences were found in POSAS scores. However, regression analysis revealed that transverse incision significantly improved satisfaction (*P* = 0.04) and appearance (*P* < 0.05). Sensory scores were significantly affected by follow-up duration (*P* < 0.001).

**Conclusion:**

Transverse incisions in ALS THA may enhance cosmetic satisfaction without compromising sensory outcomes. These findings support the potential role of personalized incision planning for improving patient-reported outcomes following THA.

Video Abstract

**Supplementary Information:**

The online version contains supplementary material available at 10.1186/s42836-025-00354-7.

## Introduction

Total hip arthroplasty (THA) is excellent for relieving pain and is considered one of the most successful surgeries of the twentieth century [1]. However, in recent years, many patients hope to achieve high levels of postoperative function, and greater emphasis is being placed on patient satisfaction [2]. Many studies investigated the factors that determine satisfaction after THA [3], and surgical incision is considered an important factor.

In the direct anterior approach (DAA), one of the anterior approaches to THA, a transverse skin incision called the bikini incision has been attracting attention [4–6]. Classical longitudinal incisions do not follow the relaxed skin tension line (RSTL), which may impair wound healing and result in poor cosmetic results. The importance of the skin incision is thought to be similar in the anterolateral supine approach (ALS) [7–9]. Compared with DAA, ALS has attracted attention because of its lower risk of lateral femoral cutaneous nerve damage and lower rate of wound complications [10–14]. However, research on transverse incisions in ALS THA is lacking, and in particular, few studies have used objective indicators of the cosmetic aspect.

The Patient Observer Scar Assessment Scale (POSAS) has been used for scar assessment after THA [15, 16]. The POSAS is a reliable method for assessing scarring and is suitable as a patient-reported outcome measure. Previous studies have used the POSAS for evaluation, but these studies excluded bikini incisions in DAA THA [15]. To our knowledge, no studies have used the POSAS to evaluate transverse incisions in ALS THA. Objective evaluation of transverse incisions in ALS may be useful for selecting incisions for each patient, which may ultimately lead to personalized THA.

The purpose of this study was to compare the differences between conventional longitudinal and transverse incisions in ALS THA. The hypothesis was that transverse incisions would improve the cosmetic appearance in comparison with longitudinal incisions, thereby increasing patient satisfaction following ALS THA.

## Methods

### Study population and design

Our institutional ethics review board approved this study. The study retrospectively analyzed 168 consecutive hips undergoing primary ALS THA between November 2019 and November 2024. During this period, surgery was performed using longitudinal incisions until December 2022 and using transverse incisions thereafter. All surgeries were performed by a single board-certified orthopedic surgeon with more than 10 years of experience.

**Fig. 1:**
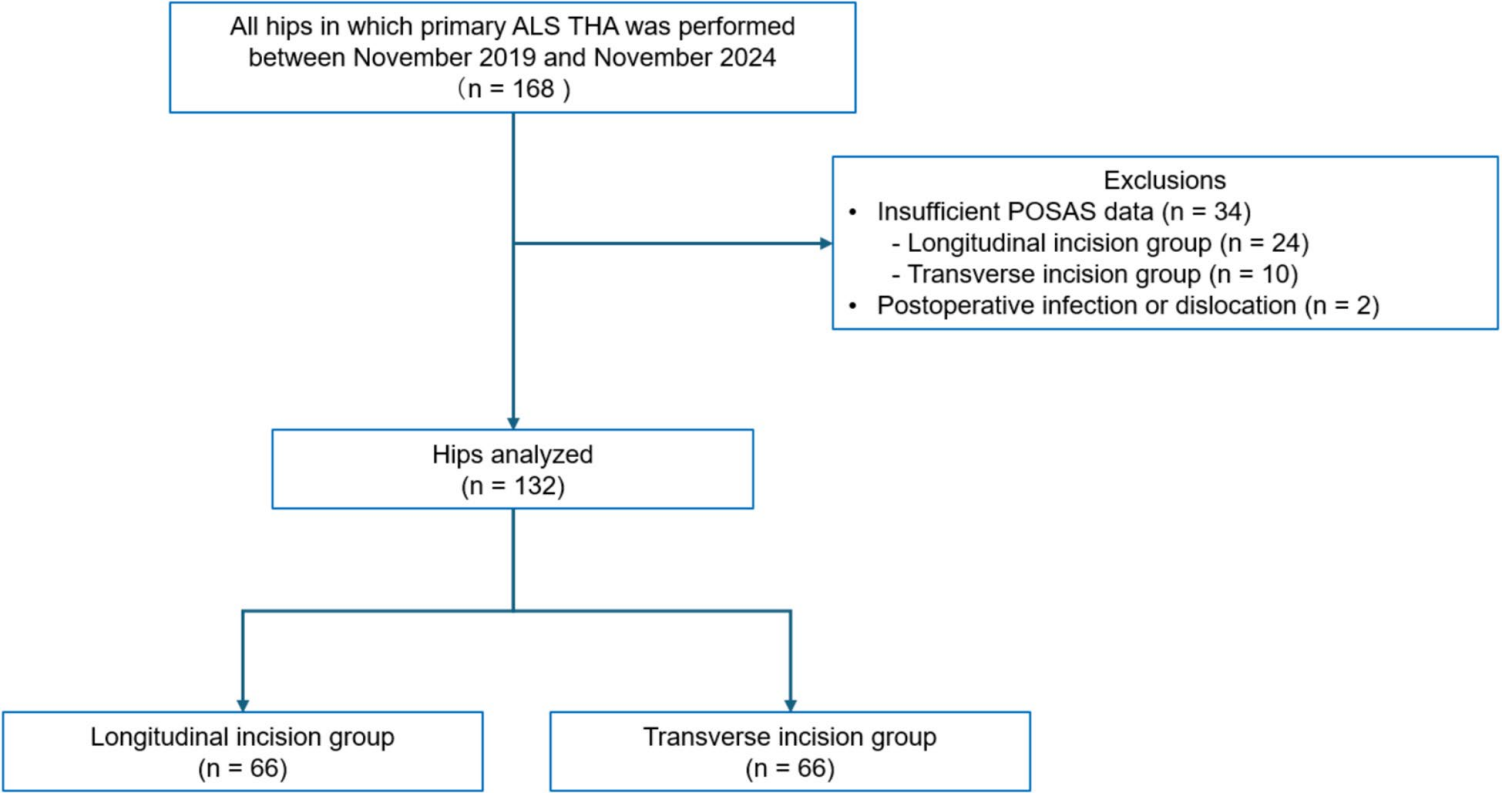
Flowchart showing the process used to select study participants. *ALS, anterolateral supine approach; POSAS, Patient Observer Scar Assessment Scale; THA, total hip arthroplasty*

The inclusion criterion was primary ALS THA. The exclusion criteria were as follows: insufficient POSAS data; additional surgery on the affected hip for infection or dislocation during the follow-up period. Therefore, after the exclusion of 46 THAs, 132 THAs were included in the analysis. Figure 1 shows a flow diagram of the study.

**Fig. 2:**
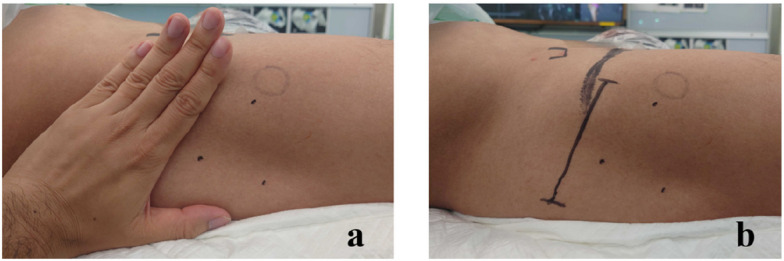
Determining the position of the transverse incision for ALS THA. A right hip intraoperative shot showing the caudal extent of the patient on the right side of the image and the anterosuperior iliac spine landmark on the left. A line parallel to the relaxed skin tension lines was placed four fingerbreadths distal to the anterior superior iliac spine, originating from the center of the tensor fascia lata muscle and extending approximately 9 cm posteriorly. *ALS, anterolateral supine approach; THA, total hip arthroplasty*

**Fig. 3:**
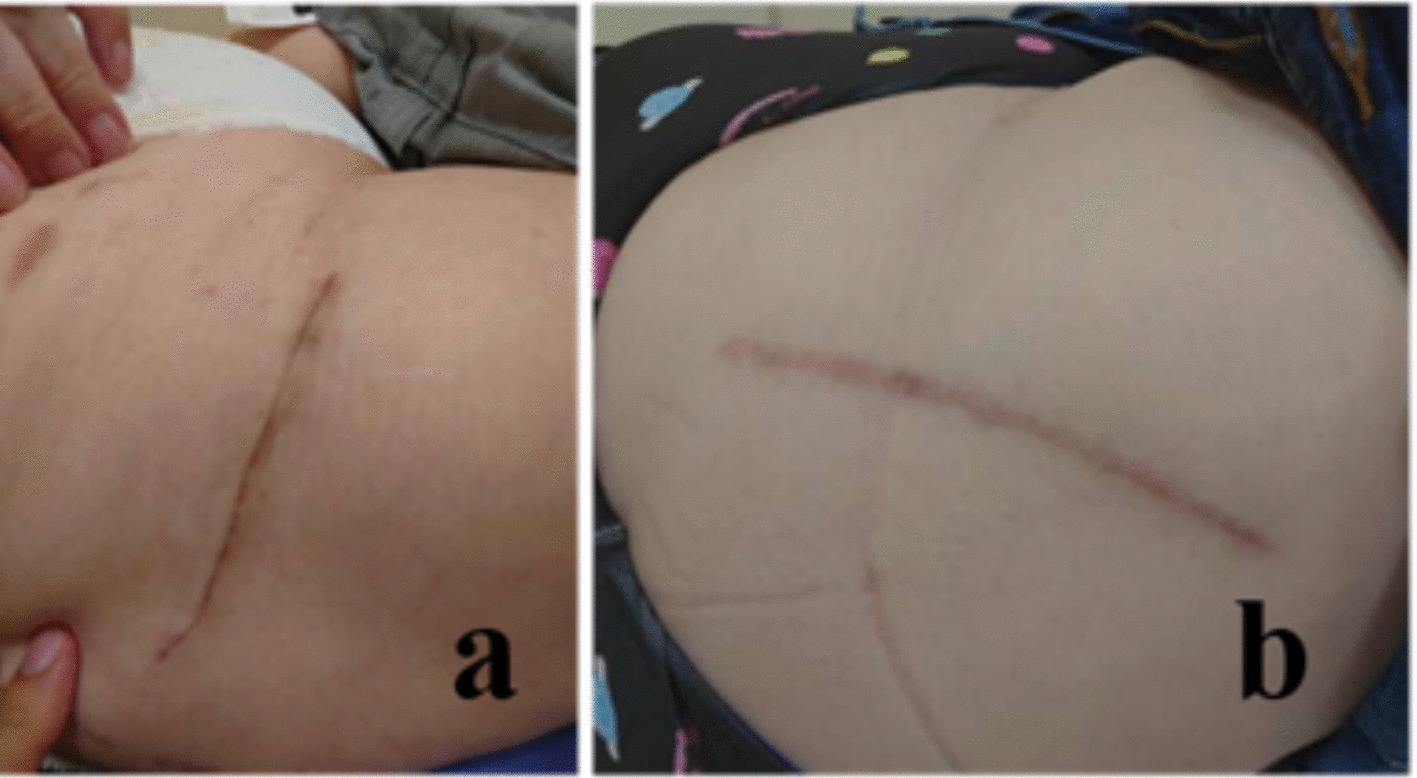
Photograph showing incisions for right-sided ALS THA. **a** Transverse incision. **b** Longitudinal incision. *ALS, anterolateral supine approach; THA, total hip arthroplasty*

The transverse incision was aligned with the RSTL, initiated at the center of the tensor fascia lata muscle, approximately four fingerbreadths distal to the anterior superior iliac spine (Fig. 2). On the other hand, the longitudinal incision was made from the middle of the greater trochanter, slightly anterior to the anterior superior iliac spine (Fig. 3). The same procedure was performed after the exposure of the fascia lata in both incisions. A V-shaped incision was made in the anterior capsule. Following the insertion of the actual components, a synthetic absorbent thread was used to suture the anterior capsule that had been cut [17]. The acetabular components primarily consisted of uncemented cups (R3, Smith & Nephew; G7, Zimmer Biomet) in all cases. Similarly, uncemented stems were selected in all cases (POLARSTEM, Smith & Nephew; SL-PLUS MIA HA, Smith & Nephew; Avenir Complete Hip system, Zimmer Biomet). Drainage tubes were not used in any cases. Subcuticular absorbable monofilament suture was used for all cases. The surface was coated with a hydrocolloid dressing.

Regardless of the skin incision method, all rehabilitation programs were uniform. On the day after surgery, full weight-bearing without range-of-motion restrictions was started.

### POSAS scale

POSAS 3.0 was used to assess the scar following ALS THA [18]. The POSAS includes two distinct scales, an observer scale and a patient scale, allowing scar quality to be assessed from both professional and patient perspectives. These items were evaluated using three categories: satisfaction, appearance, and sensory. The satisfaction category was scored out of 5, with 5 representing the greatest satisfaction. The appearance and sensory categories were scored out of 30 and 55, respectively, with lower scores representing better outcomes. The POSAS was checked at 6 and 12 months postoperatively, and annually thereafter. The score at the final observation was used.

### Statistical analysis

Descriptive statistics are presented as the median (range), number (percentage), or mean and standard deviation, as appropriate. The Shapiro–Wilk test was used to assess the data for normality of distribution. Differences between the transverse incision group and the longitudinal incision group were evaluated using the chi-squared test for categorical variables and the Mann–Whitney *U* test for continuous variables. The primary outcome was the POSAS score. Multiple regression analysis was conducted, with each POSAS subscale used as a response variable. Explanatory variables included sex, body mass index (BMI), type of incision, and postoperative follow-up period.

All statistical analyses were performed using EZR (Saitama Medical Center, Jichi Medical University, Tochigi, Japan). A *P*-value of < 0.05 was considered statistically significant.

## Results

### Baseline characteristics

Baseline demographic characteristics were similar between the transverse and longitudinal groups (Table 1). The mean age was 64.7 ± 11.0 years in the transverse group and 61.7 ± 11.6 years in the longitudinal group (*P* > 0.05). The proportion of male patients was 24.2% in the transverse group and 25.7% in the longitudinal group (*P* > 0.05). BMI was 24.6 ± 3.6 in the transverse group and 24.6 ± 4.5 in the longitudinal group (*P* > 0.05). However, there were significant differences between the two groups in the follow-up period and skin incision length. The mean follow-up period was 12.8 ± 5.9 months in the transverse group and 41.2 ± 12.5 months in the longitudinal group (*P* < 0.0001). The mean incision length was 9.3 ± 1.2 cm in the transverse group and 11.8 ± 0.6 cm in the longitudinal group (*P* < 0.0001).

**Table 1:** Demographic data and POSAS scores

Parameter	Longitudinal incision (*n* = 66)	Transverse incision (*n* = 66)	*P*-value
Age at surgery, years, mean ± SD, range	61.7 ± 11.6 [27–82]	64.7 ± 11.0 [38–87]	n.s
Male sex, *n* (%)	17 (25.7)	16 (24.2)	n.s
BMI, kg/m^*2*^, mean ± SD, range	24.6 ± 4.5 [17.3–35.5]	24.6 ± 3.6 [19.0–39.0]	n.s
Follow-up period, months, mean ± SD, range	41.2 ± 12.5 [24.2–61.4]	12.8 ± 5.9 [5.0–24.3]	**< 0.0001**
Incision length, cm, mean ± SD, range	11.8 ± 0.6 [10.0–12.0]	9.3 ± 1.2 [7.5–12.0]	**< 0.0001**
POSAS satisfaction, mean ± SD, range	3.9 ± 0.8 [2–5]	4.1 ± 0.9 [2–5]	n.s
POSAS appearance, mean ± SD, range	11.2 ± 4.7 [6–23]	11.4 ± 4.1 [6–24]	n.s
POSAS sensory, mean ± SD, range	13.0 ± 3.9 [11–33]	13.3 ± 3.3 [11–23]	n.s

*BMI* body mass index, *POSAS* Patient Observer Scar Assessment Scalem *SD* standard deviation, *n.s*. not significant

### POSAS evaluation

There were no significant differences between the two groups in any subscale of the POSAS.

**Fig. 4:**
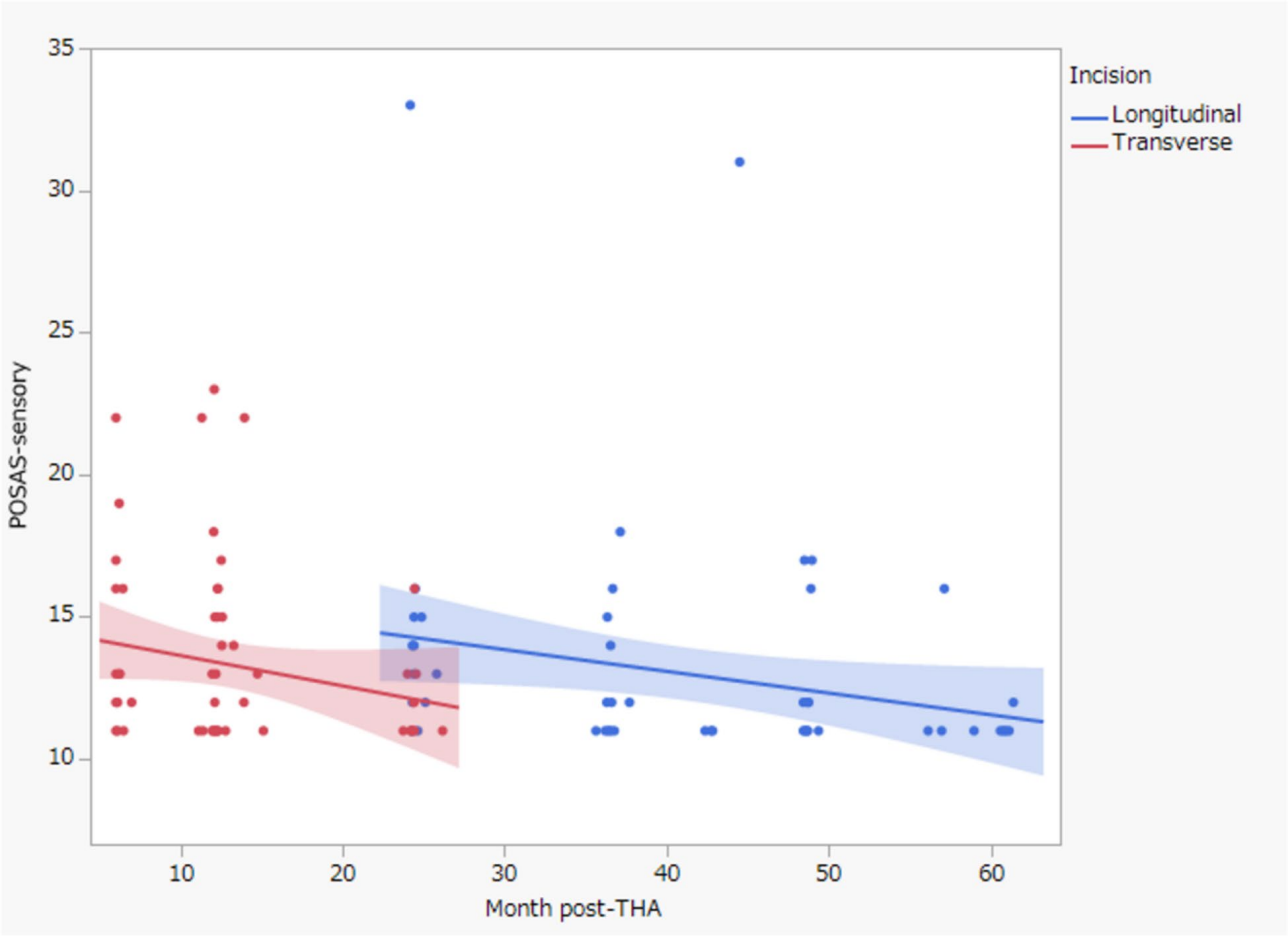
Relationship between postoperative progress and POSAS sensory category. The solid line represents the transverse incision, and the dotted line represents the longitudinal incision. *POSAS, Patient Observer Scar Assessment Scale; THA, total hip arthroplasty*

The results of the multiple regression analysis are shown in Tables 2, 3 and 4. Transverse incision was identified as a significant variable for the satisfaction category (*P* = 0.04). Transverse incision, BMI, and follow-up period were identified as significant variables for the appearance category (*P* < 0*.*05). Finally, the follow-up period was identified as a significant variable for the sensory category (*P* < 0*.*001). Figure 4 shows the gradual improvement in sensory scores over the postoperative course following THA, with the transverse incision demonstrating superior scores at around 24 months postoperatively.

**Table 2:** Results of multiple regression analysis for the satisfaction category

Variables	Estimated regression coefficients (β)	Standard error	*t*-value	*P*-value
Male sex	-	-	-	-
BMI	-	-	-	-
Transverse incision	0.58	0.28	2.03	**0.04**
Follow-up period	-	-	-	-

*BMI* body mass index

**Table 3:** Results of multiple regression analysis for the appearance category

Variables	Estimated regression coefficients (β)	Standard error	*t*-value	*P*-value
Male sex	-	-	-	-
BMI	−0.23	0.09	−2.57	**0.01**
Transverse incision	−2.65	1.29	−2.04	**0.04**
Follow-up period	−0.1	0.04	−2.72	** < 0.01**

*BMI* body mass index

**Table 4:** Results of multiple regression analysis for the sensory category

Variables	Estimated regression coefficients (β)	Standard error	*t*-value	*P*-value
Male sex	-	-	-	-
BMI	-	-	-	-
Transverse incision	-	-	-	-
Follow-up period	−0.08	0.03	−2.63	**<0.01**

*BMI* body mass index

## Discussion

The most important finding of this study was that the choice of transverse incision contributed to the satisfaction and appearance categories of the POSAS. On the other hand, the lack of contribution to sensory outcomes was considered to be due to the fact that sensory disturbances often resolve shortly after surgery [13, 14]. Considering the findings of this study, transverse incision may be useful in ALS THA, similar to the bikini incision in DAA THA.

The impact of transverse incisions on satisfaction and appearance scores was considered to be associated with the presence or absence of scar formation at the wound site. In the transverse incision, the skin was incised along the RSTL, which is believed to reduce excessive wound tension following THA [4, 5, 12]. Quantitatively evaluating hypertrophic scars remains challenging. Further research is required to determine the incidence of hypertrophic scarring.

One notable advantage of the POSAS is its simplicity and non-invasive nature. Additionally, it enables a comprehensive assessment of qualitative scar characteristics, including vascularity, pigmentation, thickness, relief, pliability, and surface irregularities [18]. However, the POSAS has several drawbacks. It primarily evaluates superficial scars, and patient-reported outcomes may be ambiguous for areas like the hip joint, which are typically covered by clothing. Moreover, observer assessments are inherently subjective, and scores may vary depending on the evaluator. However, this issue may have been mitigated in the present study, as all evaluations were conducted by a single surgeon. Additionally, the POSAS is limited in its ability to monitor long-term changes, making it difficult to quantitatively track scar evolution over time.

A strength of this study lies in its direct comparison of two types of incisions performed by a single surgeon. A previous report compared transverse incision and longitudinal incision in DAA THA at the same facility [5]. A previous study by Leunig et al. [5], which analyzed just under 1,000 THAs at a single institution, raised concerns due to the involvement of six different surgeons. An advantage of involving a single surgeon is the reduction of procedural variability. Consistency in surgical technique enhances the internal validity of the findings, enabling greater confidence that the outcomes are attributable to the intervention itself rather than to inter-surgeon differences.

The difference in incision length between the two groups was a notable finding (Table 1). The average skin incision length for the DAA THA is reported to be 6–8 cm [12], which was considered comparable to the average transverse incision length of 9 cm reported in this study. Transverse incisions tended to follow natural skin tension lines, resulting in shorter scars [12, 19].

Although the follow-up periods differed between the two groups in this study, it was noteworthy that the transverse incision group, despite having a shorter follow-up duration, exhibited superior sensory scores, which typically improve over time [13, 14]. This suggests that further improvements may be observed in the transverse incision group if the follow-up period is extended. This difference may be attributable to lateral femoral cutaneous nerve damage. Further investigation is essential to validate these findings.

Despite the strengths of this study, certain limitations should be acknowledged. Most notably, clinical outcomes beyond those assessed by the POSAS were not evaluated. Specifically, radiographic parameters such as cup placement angles and patient-reported outcome measures, including the Hip Disability and Osteoarthritis Outcome Score and the Forgotten Joint Score, were not incorporated [20]. Furthermore, the POSAS comprises an observer scale and a patient scale; considering that the same surgeon administered the survey, the possibility of bias remains a concern. Second, significant differences in the follow-up period were observed between the groups. A longer follow-up duration may influence outcomes related to transverse incision. Further investigation is warranted. In addition, although the sample size was relatively small, it was comparable to that of previous studies using the POSAS to assess scars after THA [15]. Furthermore, 34 of the 168 patients were excluded due to incomplete POSAS data. The reduced score collection rate observed in the longitudinal incision group is likely a consequence of the extended follow-up duration, which resulted in missed evaluations owing to patient attrition or infrequent outpatient visits. Finally, the significantly shorter length of the transverse incisions may independently influence aesthetic outcomes and patient satisfaction.

## Conclusion

Transverse incisions in ALS THA improve cosmetic outcomes and patient satisfaction without compromising sensory perception. This study was the first to evaluate ALS THA using the POSAS, and the results support the use of transverse incisions as an alternative to traditional longitudinal incisions in ALS THA.

## Data Availability

The datasets used and/or analyzed during the current study are available from the corresponding author upon reasonable request.

## Abbreviations

ALS: Anterolateral supine
BMI: Body mass index
DAA: Direct anterior approach
POSAS: Patient Observer Scar Assessment Scale
RSTL: Relaxed skin tension line
THA: Total hip arthroplasty
